# Clinical nurses’ awareness and caring experiences for patients with cervical cancer: A qualitative study

**DOI:** 10.1371/journal.pone.0217201

**Published:** 2019-05-21

**Authors:** Hae Won Kim, Duck Hee Kim, Yeon Hee Kim, Eun Ju Lee, Saem Yi Kang, Da Bit Lee, Youngji Kim

**Affiliations:** 1 Research Institute of Nursing Science, College of Nursing, Seoul National University, Seoul, Republic of Korea; 2 Department of Nursing, Woosuk University, Wanju, Republic of Korea; 3 Department of Clinical Nursing, University of Ulsan, Ulsan, Republic of Korea; 4 College of Nursing, Seoul National University, Seoul, Republic of Korea; 5 Department of Nursing, College of Nursing and Health, Kongju National University, Gongju, Republic of Korea; University of Auckland, NEW ZEALAND

## Abstract

To determine the degree to which nurses are aware of cervical cancer and to describe nurses' experiences of caring for patients with cervical cancer. To promote quality of nursing care of cervical cancer, we need to explore their perceptions and nursing experience in doing cervical-cancer care. This study was a qualitative descriptive design. Interviews were conducted with 14 registered nurses. The interviews were audiotaped, transcribed and analyzed. Content analysis was performed. Fourteen nurses who had been working at wards and cancer education centers were recruited in this study. Nine key themes emerged from three categories such as nurses’ awareness of cervical cancer, awareness of cervical cancer patient and caring experience. Nurses expressed fear of cervical cancer and helplessness in the face of a life-threatening prognosis. Nurses stated that they might have prejudice about cervical cancer, since it is caused by a sexually transmitted disease. They also recalled that patients with cervical cancer were more sensitive and demanding. Our findings provide a comprehensive and in-depth perspective in understanding the experience of caring for cervical cancer patients. Clinical nurses showed complex emotional reactions to cervical cancer, and expressed prejudice against the sex life of cervical-cancer patients. More education is required to ensure that clinical nurses can provide a nurse-led intervention with patients by managing nurses’ fear, prejudice, and the care burden.

## Introduction

Cervical cancer is a fatal disease in women worldwide. Globally, 270,000 people die from cervical cancer each year, necessitating further efforts for its treatment and prevention [1]. In Korea, where the incidence and mortality rate of cervical cancer are 6^th^ and 9^th^, respectively, among women’s cancers, about 3,500 new cases are reported every year, about 900 of which lead to death, rendering cervical cancer a serious health problem [2].

The standard treatments of cervical cancer are concurrent chemo radiotherapy (CCRT) and surgery [3]. The survival rate from cervical cancer has increased with the development of therapies, and cancer survivorship care issues are increasing. Cervical-cancer patients experience multiple symptoms and side effects, depending on the stage and the patient’s status. Nursing intervention should be provided for the individual in a customized way [4]. From cancer diagnosis to end of treatment, the role of oncology nurses is essential to caring for cancer patients during the disease trajectory.

Health professionals have perceived barriers to cervical-cancer care, such as lack of awareness of cervical cancer, and inadequate knowledge of and skills in nursing care [5,6]. Health professionals’ misconceptions about the disease and lack of knowledge have led to suboptimal care due to insufficient information being given to patients [7]. Nurses’ experiences with practical knowledge affect the nursing process. Expert nurses with practical knowledge have more positive attitudes toward patients than do less experienced nurses [8]. Nurses’ perceptions about cancer are reflected in cancer-patient care, and are closely related to providing optimal nursing care. To strengthen the role of oncology nurses, we need to explore their perceptions and nursing experience in providing cervical cancer care. However, little is known about nursing experience and nurses’ perspectives on cervical cancer and patient care. Also, current nursing research in the cervical cancer area has focused on disease prevention and early protection rather than on nursing care [9,10].

In recent research, compared to ovarian cancer, doctors used several negative words and showed prejudice towards cervical cancer [11]. However, we were unable to find a study on nurses’ awareness of cervical cancer or cervical cancer patients. We thus need to determine whether nurses are prejudiced toward cervical cancer, and if so, whether this prejudice affects their nursing practice.

Thus, this study describes nurses’ perception and experiences in caring for patients with cervical cancer. We hope this study will provide valuable data for nursing intervention and professional nursing care for patients with cervical cancer.

## Materials and methods

### Design

This study uses narrative and qualitative research to determine awareness of participants, in order to explore the experience of nurses who care for cervical cancer patients.

### Sample and recruitment

Convenience sampling was employed to select participants from general hospitals in Seoul and Gyeonggi-Do. We visited several nursing departments in hospitals to explain the purpose of the study, and asked for cooperation. We were introduced by the nursing department to nurses who met the criteria for selection (nurses who had been working at wards and outpatient departments of gynecology or cancer education centers for more than three years). Of these, 14 nurses who wanted to participate in the research were selected as participants. Twelve nurses were working at inpatient units and two nurses were working at a cancer education center.

### Data collection

Data were collected from April 27 to August 26, 2016, using one-on-one in-depth interviews. The interviews took place either in the hospital lounge or in an empty office, where we could maintain privacy for their convenience. The interviews were conducted before or after working hours, and took about 30 minutes to 1 hour. Each participant was interviewed at least once. The interviews were conducted by the primary investigator and co-investigator and the data were collected until no new information or relevant data arose. The interviews started comfortably and naturally with a daily greeting. Key themes emerging during the interviews were investigated in more depth through further questioning. Open-ended questions were asked to enable participants to freely express their experiences and thoughts. The main questions for the interview were:

What do you think about cervical cancer?
What do you think about patients with cervical cancer?
What was your experience in caring for a woman with cervical cancer and her family?
What did you feel while nursing?
What were the difficulties while nursing?
Has the experience of nursing women with cervical cancer affected your own nursing behavior?

In order to collect accurate data, we voice-recorded interviews with the consent of the participants, and recorded their responses using field notes. After the interview, the researcher listened to the recordings many times until they could be transcribed verbatim. In writing the document, questionable or ambiguous contents were clarified by questioning the participants via telephone contact, or at the next interview.

### Data analysis

Data were analyzed using content analysis, in which a systematic and objective method is used to describe a particular phenomenon [12]. The specific procedures are as follows.

We repeatedly read all the transcripts to understand the overall meaning of the cognition and understanding of nurses caring for cervical cancer patients, and to familiarize ourselves with the data. After identifying meaningful and important sentences based on the research questions, the sentences were categorized in terms of criteria, such as nurses’ feelings, perceptions, and experience. Various subjects related to these categories were recorded and organized into subcategories.

To integrate the research results, the above process was repeated several times to rearrange the categories and topics, creating topics that were a common theme among all participants.

### Trustworthiness of the findings

The trustworthiness of the analysis was established by applying a rich presentation of the findings with appropriate quotations, and a discussion of the interpretation and reflections.

We previously conducted several qualitative studies on women’s health in nursing, and have discussed our findings with two professors of nursing who have had extensive experience in qualitative research in order to confirm the credibility of the research. To also confirm the transferability of the research, the interview contents and data analysis results were shared and verified by telephone conversations with the research participants, even after the interview. To enable confirmation of the study, a professor of nursing who had experience with qualitative research reviewed the research procedure and study results. Codes and categories were cross-checked between researchers.

### Ethical considerations

This study was approved by the Seoul National University Institutional Review Board (IRB) (IRB No. 1604/001-002), before collecting data. Before the interviews, participants were given sufficient explanations about the purpose of the study and research, the participation process, and the time required, and were informed that they could change their decision at any time during the process. In addition, we explained to the participants that the data collected would only be used for research purposes and that the confidentiality of personal information would be guaranteed. After signing the consent form for participation in the research and recording, the participants were interviewed, and given a small gratuity for participating in the research.

## Results

The participants included 14 nurses (19 were approached and 5 declined with reasons of no time or no interest), whose average age was 30.6 years (Table 1).

**Table 1:** Characteristics of the subjects (N = 14).

Characteristics	Categories	N (%)	Mean ± SD
Age			30.6 ± 5.2
	21–25	3 (21.4)	
	26–30	4 (28.6)	
	31–35	4 (28.6)	
	36–40	3 (21.4)	
Marital status			-
	Unmarried	7 (50.0)	
	Married	7 (50.0)	
Religion			-
	No	7 (50.0)	
	Buddhism	1 (7.1)	
	Christian	6 (42.9)	
Education level			-
	Bachelor	12 (85.9)	
	Master	2 (14.3)	
Economic status			-
	Low	-	
	Middle	13 (92.9)	
	High	1 (7.1)	
Have a daughter			-
	No	12 (85.7)	
	Yes	2 (14.3)	
Previously diagnosed with cervical cancer			-
	No	14 (100.0)	
	Yes	-	
Diagnosis of cervical cancer among family members			-
	No	14 (100.0)	
	Yes	-	
HPV vaccination			-
	No	5 (35.7)	
	Yes	9 (64.3)	

This research revealed that nurses expressed fear of cervical cancer and helplessness because of the life-threatening prognosis. As cervical cancer is caused by sexually transmitted disease, nurses were prejudiced about the sex life of the patients and their sexual partners. They recognized that the sexual partner’s participation in care was insufficient, and they attempted to prevent theiro prejudice from being reflected in their nursing practice. They also recognized the need for specialized nursing care that reflects the characteristics of cervical-cancer patients, while understanding the patient’s sensitivity. While they felt sympathy for the patient, they were also burdened by the sensitive condition of the patient and tended to offer nonspecific care. The nurses became more aware of the importance of cervical-cancer prevention, but they also had some optimistic reactions to their own prevention practices. Table 2 describes in more detail the conceptual framework in which nurses are specifically aware of the characteristics of cervical cancer, patients, and care.

**Table 2:** Awareness of cervical cancer and nurses’ caring experience about their cervical cancer patients.

Category	Theme
Awareness of cervical cancer	Fear of disease itself
	Helplessness due to a terrible prognosis
	Optimistic bias: this is not about me
Nurses’ awareness on patients with cervical cancer	Prejudice of cervical cancer caused by sex
	Sympathy toward patients
	A sensitive, burdened patient
Actual caring experience of nurses during caring cervical cancer patients	Nonspecific care at the hospital ward
	Trying to exclude prejudice about cervical cancer patients
	Invisible husband at the ward or patient side
	Ascertaining of awareness about cervical cancer prevention
	Recommendation for future nursing care

### Nurses’ awareness of cervical cancer

#### Fear of the disease

Nurses expressed the difficulty they encountered watching patients undergoing a painful experience in fighting against cervical cancer. This incited them to have negative ideas about the prognosis of the disease, and recognized it as a fearful disease

*Watching patients with cervical cancer, I thought the disease itself was a very dangerous cancer*. *I was somehow scared a lot. (Respondent 1)*

*I think it is a very dangerous disease, and the vaccine is enough to prevent it*. *It is a little unfortunate that I have seen a lot of people who are not good at self-care. (Respondent 8)*

#### Helplessness because of a life-threatening prognosis

The nurses expressed helpless while nursing patients with cervical cancer, as they considered it to be difficult to provide customized nursing care to meet patients’ different conditions.

*They were not in good condition, and died in pain, not a peaceful death*. *Watching them, I thought it was one of the most painful cancers… I have hardly seen anyone who got cured from the disease except those in an early stage of cervical cancer. (Respondent 5)*

*I thought I emotionally comforted them well, but the next day, the same things happened… It’s like all my efforts were in vain*. *I often felt that way. They do not complain that much… It is like I am just not comfortable treating them a little. I have to be careful about my words as well. (Respondent 13)*

#### Optimistic bias: This is not about me

Some respondents thought that vaccination was the best way to prevent the disease, but because of their prejudice—that cervical cancer was mainly caused by indiscreet sexual relations—they tended to think that they don’t need HPV vaccination.

*I thought I must get the shot, and I was scared. But the thought is … I am not that kind of person who has sexual relations with many partners, and I thought this could not happen to me*. *After I started to work at a hospital, I thought again I should get the vaccine, because I have seen many serious cases. But when it comes to the route of virus infection, I am not that kind of a person who has many sexual relationships, and I tend to think that I don’t have such risks. (Respondent 3)*

*Maybe this could happen to me? I sometimes think that I can have the disease um … in my 40s and 50s. But I just dismiss the thought (laugh), like this will not happen to me! (laugh) I just think that I should get checkups regularly*. *(Respondent 11)*

### Nurses’ awareness of patients with cervical cancer

#### Prejudice about cervical cancer as it is caused by a sexually transmitted disease

Those who nursed patients with cervical cancer had medical knowledge about the cause of cervical cancer. They therefore carefully answered this question, starting with the premise, “it could be a prejudice”. They believed that the patients might have had many sexual experiences or that they had contracted the disease from their sexual partners.

*Among those with cervical cancer, not all of them, four and five patients who I have recently nursed had tattoos, and many of them smoked cigarettes*. *This is just my prejudice. Such things make us have a stronger bias. (Respondent 4)*

*Until recently, many people have a prejudice that people who have many sexual contacts or multiple partners get cervical cancer… Some people indeed look like they have many sexual partners. I came to think that I still have such a prejudice*. *But I think I view those with ovarian cancer or endometrial cancer in different ways. (Respondent 5)*

Because of the prejudice that the patients’ sexual partners had caused the cervical cancer, nurses expressed their uncomfortable feelings toward the sexual partners of cervical-cancer patients, motivated by their compassion for their patients.

*Whenever I see their family, I feel… somehow sorry for them, but not their husbands… I don’t know why; (laugh) I might be overreacting*. *Yes… Because of that (the cause of the disease) I don’t think I can view their husbands in a favorable light… (Respondent 12)*

*Of course, it was not 100% their husbands’ fault, but still it is attributable to their husbands to some extent, so I felt that women should choose partners well for marriage*. *(laugh) I came to think that way… Just um… I couldn’t help but keep looking at their husbands. (Respondent 12)*

#### Sympathy toward patients

Those who had nursed patients of a similar age to themselves or who had a young child tended to have sympathy and compassion for their patients, and deeply sympathized with the difficulties patients underwent as “women”, considering this could easily also be their experience.

*I have seen young mothers who were diagnosed with the disease when their child was young*. *They were diagnosed when they got a checkup after having gotten pregnant or given birth. That makes me think of my child and worry about how their family members feel. For such reasons, I started to feel compassion for them, and I don’t think because they did something bad or other things, they got the disease. This can happen to anyone, so I sometimes think that this kind of thing could happen to me someday. (Respondent 11)*

*I tried to converse a little more with them… yes… I just tried to check if there was anything uncomfortable*, *not about their private life or history… whether they have any pain… I was kind of more worried about them and cared for them a little more? I feel sorry for them… (Respondent 12)*

Nurses generally said that, while nursing patients with cervical cancer, they noticed many patients became helpless and desperate, and that the patients’ negative emotions affected their own will to treat them, so they experienced many depressing episodes. While the nurses felt sympathy for the patient’s helplessness, they also felt helpless themselves because of the life-threatening cervical cancer prognosis.

*In fact, they seemed to have little intent to have active treatment … This is my judgment*. *It looks a bit less than other cancers. They seem to give up a little. (Respondent 4)*

*I feel like they’re surrounded by more negative things now … I felt a little bit*. *Patients with ovarian cancer are more positive and optimistic? They seemed to be a little bit … Can it be depressed? (Respondent 14)*

#### The more sensitive and burdened patient

Respondents stated that patients with cervical cancer tend to be sensitive, regardless of their age. Recalling their experiences of nursing patients with various types of gynecologic cancer, they expressed that patients with cervical cancer were more sensitive and demanding, although this could have reflected their subjective opinion.

*Many of them were so picky, demanding a lot and unusual… There were so many people like that*. *Anyway, it was quite difficult to meet their demands. They were so unusual. (Respondent 5)*

*So I felt that those with cervical cancer were a little sensitive. Compared to those with ovarian cancer, um … they were … more distrustful … they asked for more explanation–about things like levels … test results … so they seemed to continue to be obsessed with negative things … I got the impression from them a lot*. *(Respondent 14)*

### Actual caring experience of nurses while caring for cervical-cancer patients

#### Nonspecific care in the hospital ward

Respondents stated that nursing services were not differentiated in terms of the distinct characteristics of cervical cancer and other diseases. Most nurses said they provided patients with cervical cancer the same post-operative nursing care as that given to other patients based on common standards, because the patients did not request any extra treatment. When asked about certain topics they thought could be sensitive, the nurses expressed that neither patients nor nurses mentioned these topics, since patients might feel uncomfortable.

*Indeed, we do not talk about the cancer that much*. *Once an operation is done, we provide standardized post-operative care for patients. We do not provide care differently or talk to guardians more because they have cervical cancer. We mostly explain general nursing procedures following operations and discuss them with patients and guardians. (Respondent 1)*

*There was no such thing as requesting more things because they had cervical cancer. There were no special requests*. *I did not pay special attention to them. For patients with cervical cancer? I haven’t given special treatment to them. I just follow the standards. (Respondent 5)*

Nurses thought that patients would be reluctant to discuss the cause of their cancer, since the patients assumed that the cause was sexual relations with sexual partners. Nurses also seemed to be uncomfortable mentioning the topic, and thus showed hesitation and avoidance.

*At first… actually there was no difference outwardly, but I tend to be careful about my words*. *Ensuring patients close their mind less? Because we feel sorry for them if they are alone. No matter whose fault it is, the one who gets the disease is the patient herself. It could be a prejudice, but I try my best not to talk about it. (Respondent 4)*

*I am not sure it was because I am a bit sensitive about it or not, but I don’t think I like to talk about it … I haven’t asked about it before, but anyway that would be very painful … I thought like that*. *I feel very sorry for them about it. (Respondent 14)*

#### Trying to exclude prejudice about patients with cervical cancer when providing care

Most respondents stated that, while they had a prejudice against patients with cervical cancer because of the cause of the disease, the prejudice did not affect their nursing service. They specifically expressed that they were professional nurses and could control their thoughts and emotions, and that such personal thoughts and feelings did not affect their service.

*This does not affect my service*. *This is just a prejudice. I just think that there might be that kind of people like that, but this does not affect nursing that much. (Respondent 4)*

*This does not affect nursing. Because I have over ten years of clinical experience—and I think that I can control my feelings in front of patients to some extent whether I feel good or bad. I think I am a pro. So even though I have such a prejudice, that is all my personal thought. When I am nursing or giving medicine to patients*, *I do not treat them in a different way. (Respondent 5)*

#### Invisible sexual partner in the ward or at the patient’s side

Among the impressions they received from the family members of cervical cancer patients, nurses more often witnessed the mothers of patients, devotedly attending to daughters, than the sexual partners. While this may be due to many reasons, respondents thought that the sexual partners treated their wives indifferently.

*Most husbands hardly came to see their wives*. *Some had never come to visit their wives, and of course there were some who had devotedly attended to their wives, but that was a rare case. Mostly the mothers of patients come to attend to patients. (Respondent 5)*

*Emotional perspective? Mothers were more likely to care for their daughters because they might feel upset, sad, and sorry for their daughters. They seemed to express such feelings more. When mothers were attending to them… (but) When husbands are attending to their wives, of course they may feel sorry, but just… they seem to feel like it can’t be helped? Um … it is more like that*. *(Respondent 12)*

#### Ascertaining awareness about cervical-cancer prevention

Respondents stated that while watching patients suffering from cervical cancer, especially those of the same age, they had considered the possibility that they or their friends or family could also contract the disease. They also stated that this awareness alerted them to the seriousness of the disease.

*Somehow, I thought vaguely that I should be “careful”, but the experience of nursing them alerted me a lot to the seriousness of the disease*. *When my friends and I had a conversation about our sexual experiences, and we heard that some of our boyfriends had sexual relations with several women, we just said like really? And we didn’t care about it that much. But these days, I started to be concerned about their health as well. (Respondent 2)*

Respondents said that they actively informed their family members and friends of information on cervical cancer accumulated from their nursing experiences, such as the contributing factors and prevention methods for cervical cancer, to ensure others as well as themselves are kept informed as a means of prevention.

#### Recommendations for future nursing care of patients with cervical cancer

The nurses believed that patients with cervical cancer might have lost their womanhood and it might affect their relationship with partners.

*Well*, *while getting treatment and after having the operation*, *they can have relations*, *but their self-esteem about womanhood may not be the same as before*. *This is not stomach cancer*, *and this is not an organ that is unrelated to sexual relations*. *I think being diagnosed with cervical cancer itself can make women think that they have lost their womanhood*. *If they have spouses*, *their spouses may give up*, *and they might also worry like*, *what if it gets worse*? *So there would be such problems between husband and wife (Respondent 3)*.Even though various education programs need to be provided in clinical fields to meet the distinct needs of cervical cancer patients, nurses were only provided with general instructions for caring for cancer patients, such as those for surgery patients or patients undergoing anticancer treatments. While nurses struggled to provide specific care for cervical cancer patients, they theoretically understood the importance of providing specialized and individualized nursing services. However, while discussing this issue, they came to understand the necessity of specialized care.

*I just came to think that such customized cervical-cervical cancer education programs might indeed need to be provided… But on the other hand, if patients know the conclusion and then approach it… Then, that could destroy their hope… like to recur…. processes like that… Patients may have already known them by searching for such information online or obtaining it from other media*. *While talking about this, I came to feel that such programs for cervical cancer or other cancers… more professional programs could be helpful. Like we do with an anticancer treatment education program, it will be better to set a certain program for it… (Respondent 11)*

## Discussion

This study was conducted to explore the experiences of nurses caring for cervical-cancer patients. To the best of our knowledge, the nurses’ feelings and perceptions of the cervical cancer presented in this study were not evident in previous studies of cervical cancer care.

Overall, nurses’ impression that cervical cancer was a terrible disease may be influenced by their practice setting. Most of them were working at inpatient units, so they would not have seen the many earlier staged woman who were cured of their cancer. The nurses expressed fear of the cervical cancer disease, as by nursing cervical cancer patients, they were aware of the seriousness of the disease. In previous studies, nurses’ fear about the illness affected passive patient care, and became a barrier preventing the nurses from treating the patient positively; the nurse’s feeling were then transmitted to the patient [13,14]. The nurses demonstrated a negative perception of cervical cancer, which might be an obstacle to providing optimal nursing care [13]. We carefully cite the possibility that the nurses in this study might not be able to actively intervene in patient relationships and patient care, because of their fear of cervical cancer.

Nurses had felt helplessness about nursing practices that seemed to have no effect on the prognosis for cervical cancer, which would generally be considered serious. This is similar to the case revealed in other qualitative studies where nurses felt helpless when experiencing patient death during the course of treatment [15,16].

Closely watching and nursing cervical cancer patients as their disease progresses is a difficult and challenging experience. Because nurses who care for cervical cancer patients have negative perceptions about the illness, such as fear and helplessness, they need psychological support and education on coping strategies [17].

In this study, the nurses were aware of the severity of cervical cancer, and were reluctant to believe that they would suffer from cervical cancer. The nurse vaccination rate was reported to be 45% for HPV (72% for physicians), demonstrating the need to increase the vaccination rate of health care personnel [18]. In this study, nine (64.3%) of the 14 nurses who participated had been vaccinated and five nurses who aged over 30 ages had not been vaccinated. Vaccination rate in our participants was higher than that in participants of other studies. In other studies, vaccination rate of HPV in nursing students and clinical nurses in Korea has been reported to be 34.2% or 20.3% [19, 20]. HPV Vaccination rates of adult married women in Korea vary by studies, from 6.1 to 19% [21, 22]. Because most of our participants were taking care of inpatient patients, it might be the reason why HPV vaccination rate was higher than that other participants. However, future study is needed to describe HPV vaccination uptake in clinical nurses.

Nurses considered the cause of cervical cancer to be a reckless sexual life and caused by sexually transmitted diseases, while some nurses tended to interpret smoking or a tattoo as negative behavior. These results are consistent with those of some previous studies [6,11, 23]. In addition, this study importantly discovered the nurses’ prejudice about the sexual life of their patients, as well as their negative feelings toward the sexual partners of the cervical-cancer patients. Such prejudices against cervical cancer patients have not been reported in cancer studies relevant to these patients [24,25]. However, nurses attempted to overlook their prejudice while caring for their cervical cancer patients. They also attempted to understand the needs of cervical cancer patients and their condition, and to nurse them, even though they had some characteristics of the sexually transmitted diseases of cervical cancer.

Nurses felt sympathy and compassion for the cervical cancer patients, as has been found in other leading studies [26,27]. In this study, our participants also expressed both prejudice and sympathy, which are common when caring for a patient with a stigmatized disease [28]. Nurses need to acknowledge such ambivalence in the nursing experience for patients with cervical cancer, and try to reduce prejudice and negative emotions. The practical nursing experiences of nurses who care for cervical-cancer patients are outlined below.

In this study, nurses were reluctant to intervene in the patients’ sexual or emotional problems, and provided general surgery nursing, instead of personalized nursing for cervical-cancer patients. Nurses said that patients felt uncomfortable sharing their sexual life or emotional problems with the medical staff, as can be seen in another study [27]. Nurses that offer general surgical nursing deliver non-customized nursing, and this many not consider the specific emotional needs of cervical-cancer patients. This result slightly differs to that from previous studies [29], in which nurses were shown to be reluctant to express intimacy with patients, and tended to focus on information about surgical operation or clinical nursing.

In Zamanzadeh et al.(2014)’s study, nurses pointed out the need to extend routine surgical nursing to personalized nursing care for cervical-cancer patients, and to involve the patient’s sexual partner in their care [29]. In this study, our participants felt that patients’ sexual partners were reluctant to engage in patient care.

Therefore, we suggest the development of a nursing education program that reflects the characteristics of cervical-cancer patients, and an educational program for cervical-cancer patients and their sexual partners. It is important to provide nurses with education programs, so they can actively nurse patients with specialized care of cervical cancer while managing their (the nurses’) fear of cervical cancer, prejudice, and the burden of patients. Second, in this study, nurses reported they seldom observed sexual partners in the ward of cervical cancer patients, compared to other gynecologic cancer patients. Therefore, we propose that patient and family education program be implemented for the relationship and to enhance the partner’s understanding of the disease. Psychological assistance is also needed for nursing staff to assist in dealing with the sensitive nature of the disease, especially from admission to discharge, beyond the results of previous studies [30], which mainly emphasized intervention to improve sexual function. Future studies will need to investigate the impact of cervical cancer on couples, and the psychological and sexual disabilities as well as the nursing needs of partners.

## Conclusions

In this study, nurses showed complex emotional reactions to cervical cancer. They expressed the view that the patients were a somewhat more difficult to care for than other patients, and that the sexual partner often did not support the care of the patients. Nurses had a prejudice against the sex life of cervical-cancer patients, but at the same time, tried to exclude this bias. The attitudes of nurses that caring for cervical-cancer patients should be no different to those when caring for other gynecologic cancer patients; however, this may have been limited by the nurses’ inability to discuss the sexual or psychological problems of cervical-cancer patients. It is important to provide education programs to nurses so that they can actively nurse patients with specialized cervical cancer and manage nurses’ fear of cervical cancer, their prejudice, and the burden of patients. In the future, nursing practices for cervical-cancer patient care should be supplemented by expanding the scope of nursing intervention, to focus on the restoration of sexuality and self-esteem. Considerations should be made for couples-oriented nursing, including nursing interventions, such as the sexual partner’s support role or couple adjustment.
